# Plasma Oxytocin Level and Sexual Dysfunction in Depressed Women Treated by Either Fluoxetine or Citalopram: a Pilot Clinical Trial

**Published:** 2018

**Authors:** Mohammad Abbasinazari, Mona Heidari-kord, Azadeh Mazaheri-Meybodi, Azadeh Eshraghi, Nima Bayati

**Affiliations:** a *Department of Clinical pharmacy, School of Pharmacy, Shahid Beheshti University of Medical Sciences, Tehran, Iran. *; b *Department of Psychosomatic, Taleghani Hospital, Shahid Beheshti University of Medical Science, Tehran, Iran. *; c *Department of Clinical Pharmacy, Faculty of Pharmacy-International Campus, Iran University of Medical Sciences, Tehran, Iran.*

**Keywords:** Sexual dysfunction, Oxytocin, Citalopram, Fluoxetine

## Abstract

Sexual dysfunction is a common cause of selective serotonin reuptake inhibitor (SSRI) withdrawal. Various studies indicate that decreased oxytocin is involved as a mechanism of delayed ejaculation induced by SSRIs. The aim of the present pilot study was to evaluate and compare sexual dysfunction and oxytocin levels in women being treated with either fluoxetine or citalopram. Thirty-nine women with the diagnosis of major depressive disorder were enrolled in the study. A baseline blood sample was collected and each participant was given either fluoxetine 20 mg/d or citalopram 20 mg/d. After 1 month, a second blood sample was collected and sexual dysfunction was evaluated via the Female Sexual Function Index (FSFI) questionnaire. Twenty-three women completed the study (12 and 11 in the fluoxetine and citalopram groups, respectively). After 1 month, the FSFI scores were 22.8 ± 7.8 and 22.5 ± 4.8 in the fluoxetine and citalopram groups, respectively. The oxytocin levels were 187.8 ± 38.8 pg/mL and 214.6 ± 23.1 pg/mL in the fluoxetine and citalopram groups, respectively. Statistical analysis did not reveal any difference in the FSFI score between the two groups after 1 month (*p* = 0.89). However, the oxytocin levels were significantly lower in the fluoxetine group than in the citalopram group (*p* = 0.05). We also observed a positive relationship between the FSFI score and oxytocin level at 1 month after starting fluoxetine or citalopram (r = 0.43, *p* = 0.04).A positive relationship between the oxytocin level and FSFI score supports the hypothesis that the oxytocin level plays a role in sexual dysfunction induced by SSRIs.

## Introduction

Today, selective serotonin reuptake inhibitors (SSRIs) are the first choice for pharmacotherapy of depression. The efficacy of SSRIs is superior to that of placebo and comparable to that of other classes of antidepressants in treating patients with major depression (1). Sexual dysfunction is a common side effect of SSRIs and may be a reason for the discontinuation of SSRIs among those who use them permanently. SSRIs can reduce libido in women and men, cause anorgasmia in women, and increase time to ejaculation in men (2-4). The reported incidence of sexual dysfunction varies widely because of differences in the methodology between studies, and the incidence of SSRI-induced sexual dysfunction ranges from approximately 15% to 80% of patients (5-7). Other antidepressant medications can cause sexual dysfunction; however, sexual dysfunction is more often secondary to SSRIs than to other antidepressants (6).

The exact mechanism of sexual disorders induced by SSRIs is unknown; however, they can induce sexual dysfunction via interactions with different hormones and neurotransmitters, including dopamine, prolactin, acetylcholine, and nitric oxide. Dopamine neurotransmission has consistently been shown to be involved in sexual arousal. In animal models, dopamine agonists decrease the ejaculatory threshold, whereas the destruction of dopaminergic neurons is associated with prolonged ejaculatory latency (8). Elevated prolactin concentrations are associated with a marked inhibition of sexual desire and performance (8). SSRIs may be the most common cause of drug-induced hyperprolactinemia (9). The findings are consistent with the hypothesis that cholinergic neurotransmission has a modulating effect when other neurotransmitter systems are involved (8). Nitric oxide is critical in the signal transduction pathway mediating the penile vascular changes required for erection (10).

Recently, the clinical relevance of oxytocin in SSRI-induced sexual dysfunction has been raised. It has been hypothesized that oxytocin should be able to positively influence the parameters of sexual function and couple interactions. Oxytocin is clearly involved in human reproduction and serves an important role in sexual arousal. The presence of oxytocin receptors on multiple organs such as female genital organs, suggests a possible preparatory role of oxytocin for the later and final phase of the copulatory process, such as ejaculation and orgasm, preparing all necessary muscular contraction and lubrication effects (11). Infusion of oxytocin antagonists into cranial vertebrae in rats had an inhibitory effect on female sexual behavior (12). In a recent trial, the effect of intranasal administration of oxytocin was evaluated in 29 healthy heterosexual couples. It was reported that women felt more relaxed and subgroups indicated a greater ability to share sexual desires or to empathize with their partners (13).

It seems that postsynaptic receptors of 5-hyroxytryptamine1A (5HT1A) have a potential role in oxytocin release. The increased serotonin concentrations induce oxytocin release via activation of 5HT1A receptors and this might compensate for the inhibitory effect of serotonin on sexual function (14). In animal studies, an inverse effect on oxytocin levels has been reported between fluoxetine and citalopram administration (15, 16). As a difference in oxytocin level may be part of the sexual dysfunction induced by SSRIs, we designed a clinical study to evaluate sexual dysfunction and oxytocin levels among women treated with either fluoxetine or citalopram.

## Methods

*Study design & Patient recruitment*

An open label clinical trial study, registered in Australian and New Zealand Registry of Clinical Trials (ACTRN12614000869673), was performed from January 2014 until January 2015 in the psychiatric clinic of Taleghani Hospital, affiliated to Shahid Beheshti University of Medical Sciences, Tehran, Iran. Ethical committee approval was obtained from Shahid Beheshti University of Medical Sciences before starting the study as per the provision of the Helsinki declaration (2000) and all patients were given their informed consent forms. The inclusion criteria were as follows: (1) women aged between 18 and 45 years with major depression diagnosed by an expert psychiatric physician based on the DMS-IV, who were candidates for administration of either fluoxetine or citalopram and (2) women who had no history of sexual dysfunction. Patients were excluded if they fulfilled any of the following criteria: (1) menopause, (2) pregnancy, (3) lactation, (4) history of any pituitary disorders, (5) receiving any medications that induce sexual dysfunction during the study or within the preceding 2 months, (6) receiving any medications, which can cause drug interactions with SSRIs during the study or within the preceding 2 months and (7) addiction to any agents.

Eligible patients who met the inclusion criteria were randomly allocated into either the fluoxetine 20 mg/day or the citalopram 20 mg/day group. If they met none of the exclusion criteria, the physiatrist instructed the patients to take their drugs for at least 1 month. Adherence to treatment was determined by counting the tablets left in the container at the end of 1 month. 

*Assessment of sexual dysfunction*

The Female Sexual Function Index (FSFI) questionnaire was used to measure the women’s sexual functioning on the basis of self-reports before and 1 month after the start of treatment. This questionnaire contains 19 items, covering female sexual arousal disorder (FSAD), hypoactive sexual desire disorder (HSDD), female sexual orgasm disorder (FSOD), dyspareunia/vaginismus (pain), and multiple sexual dysfunctions. Then, all scores of 6 items are added to create a total score for each patient. The total score ranges from a minimum score of 6 to a high score of 36 (17). The FSFI questionnaire has been previously validated by Mohammadi *et al*. among Iranian women to assess sexual dysfunction (18). 

*Measurement of oxytocin level*

Venous blood samples were collected from all women who met the inclusion criteria at baseline (before taking citalopram or fluoxetine), and 1 month after treatment. Samples were collected and centrifuged for 10 min within a few hours of sampling. Plasma was removed and frozen at −70 °C until completion of the study. All samples were measured in a single assay. Plasma oxytocin levels were determined by radioimmunoassay kit (Phoenix Pharmaceuticals, Inc., USA). The sensitivity was 0.6 pg/mL, and the intra- and inter-assay coefficients of variation were 7% and 15%, respectively.

*Statistical analyses*

The data were analyzed using SPSS version 20 for windows (SPSS Inc., Chicago, IL, USA) software. Mean (SD) and median (IQ) of continuous variables were compared between two groups using an independent sample *t*-test (for normally distributed data) and a Mann–Whitney U test (for non-normally distributed data), respectively. Pearson’s correlation was used to show the strength and direction of a relationship between two variables when at least one variable was normally distributed. If the assumption of normality was not met, Spearman’s rank correlation was used. *p* ≤ 0.05 was considered to indicate statistical significance.

## Results

*Characteristic of the participants*

**Figure 1:**
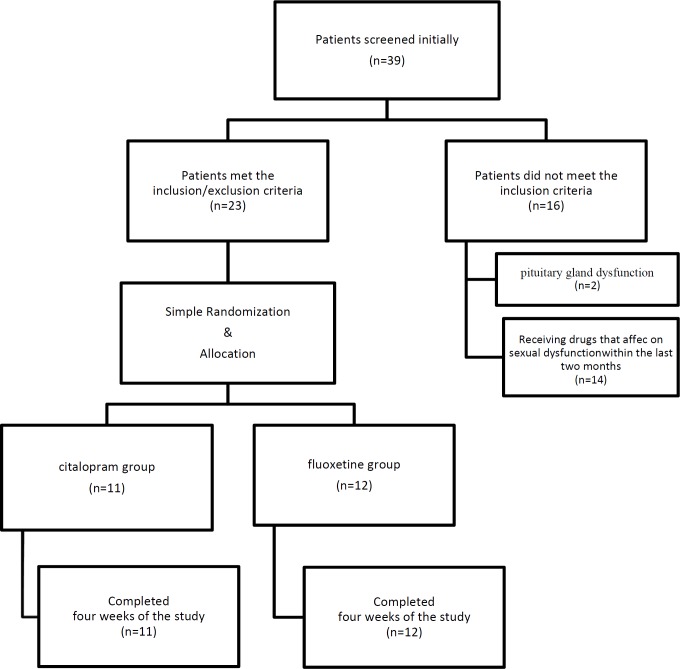
Fellow of the study

During the study period, 39 women were screened initially and finally, 23 women with an average age of 29.0 ± 7.4 years met the inclusion criteria and were admitted to the study (Figure1).Characteristics of the studied patients are shown in Table 1. Among them, 12 patients received fluoxetine and 11 received citalopram. All patients who entered completed the study until the end. 

The average ages of the study participants are shown in table 1 and statistical analysis did not detect a significant difference between the two groups (*p* = 0.41).

*FSFI scores before and after receiving medications*

The mean FSFI score of all 23 patients was 31.0 ± 2.0 before starting the medications. The FSFI scores in the two groups are shown in table 1. Statistical analysis did not detect any difference regarding baseline FSFI in the two groups (*p* = 0.89). We found a total FSFI score of 22.6 ± 6.4 after 1 month of drug treatment in all women. Average FSFI scores in fluoxetine and citalopram groups are shown in table 1. Statistical analysis did not detect significant differences between FSFI scores of patients who received fluoxetine and those receiving citalopram after 1 month (*p* = 0.89).

*Plasma levels of oxytocin before and after receiving medications*

**Figure 2:**
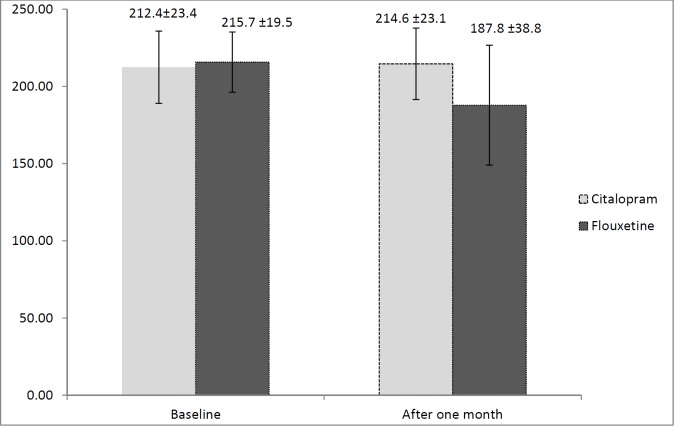
Oxytocin concentration before and after taking either fluoxetine or citalopram

Plasma levels of oxytocin before and after starting either fluoxetine or citalopram were determined and are presented in Figure 2. No significant difference was detected in oxytocin concentration between the two groups at baseline (*p* = 0.71). Mean oxytocin level was higher in the citalopram group (214.6 ± 23.1 pg/mL) than in the fluoxetine group (187.8 ± 38.8 pg/mL) after 1 month. A statistically significant difference in the mean oxytocin level was observed after 1 month between the groups (*p* = 0.05).

*Correlation between FSFI score and oxytocin level after 1 month*

**Figure 3:**
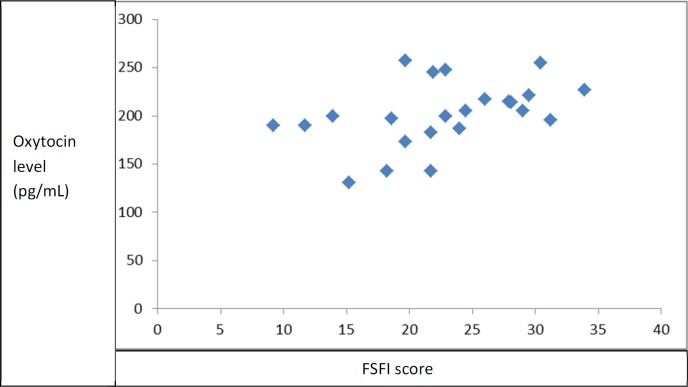
Scatterplot of FSFI score and oxytocin level

Figure 3 shows the relationship between FSFI score and oxytocin level in the study participants 1 month after starting the medications. There was a meaningful correlation found between FSFI and oxytocin level (r = 0.43, *p* = 0.04). Otherwise, there appeared to be a modest correlation between FSFI score and oxytocin level in the study participants.

## Discussion

Although there are controversies regarding the clinical role of oxytocin in sexual dysfunction, it has been mentioned as an interactive parameter of sexual dysfunction. Muin *et al.* have evaluated the use of intranasal oxytocin (32 IU) or placebo in women within 50 min before sexual intercourse. 

The primary outcome of their study was FSFI score and they concluded that following administration of oxytocin and placebo, the FSFI score increased by 26% and 31%, respectively, but no significant difference was found between the two groups (19). However, Behnia *et al*. evaluated the acute effects of intranasal oxytocin (24 IU) on sexual drive, arousal, orgasm, and refractory aspects of sexual behavior together with partner interactions. They reported that women felt more relaxed and subgroups indicated better abilities to share sexual desires or to empathize with their partners (13). Sexual dysfunction due to SSRIs appears to depend on not only on serotonin receptors but also on other mechanisms. Moreover, different hormones and neurotransmitters are involved in the development of this side effect (20). Ozsoy *et al.* have evaluated serum oxytocin levels in 40 patients with depression before and after treatment with antidepressant drugs or electroconvulsive therapy. They reported that antidepressant treatments appeared to have no effect on serum oxytocin concentration. They did not indicate which type of antidepressant agents were taken by the evaluated patients (21). Keating *et al*. evaluated plasma oxytocin in patients diagnosed with major depressive disorders before and following treatment with SSRIs. They failed to detect a difference in oxytocin concentration before and after SSRI treatment, and there were no significant relationships between oxytocin and psychological symptom scores, before or 12 weeks after treatment (22). They did not indicate which kind of SSRIs were used in the patients they evaluated.

**Table 1:** comparing of age, BMI, education level and FSFI at baseline and after 1 month between two groups.

	**Fluoxetine group** **(n = 12)**	**Citalopram group** **(n = 11)**	***p*** ** value**
Mean age (± SD)	30.4 + 7.6	27.8+ 7.3	0.41
BMI (±SD)	22.0 ± 2.2	22.7 ± 2.1	0.49
**Education level**			
Under Diploma	1	2	
Diploma	7	6	0.77
Above Diploma	4	3	
Baseline FSFI score ( ± SD)	30.0 ± 2.0	32.0± 2.0	0.89
FSFI score after 1 month ( ± SD)	22.8 ± 7.8	22.5 ± 4.8	0.89

Regarding the observation that the prevalence of sexual dysfunction is high among SSRI users, and that it is not exactly known how SSRIs impair sexual function, we designed a comparative study to measure oxytocin concentrations in women who received citalopram or fluoxetine after 1 month. We choose these two SSRIs because, in animal studies, fluoxetine and citalopram have been reported to affect oxytocin levels in opposite ways (15, 16). De Jong *et al*. have concluded that if chronic treatment with citalopram increases oxytocinergic neurotransmission whereas chronic treatment with fluoxetine inhibits it; this could underlie the difference in effect of these two SSRIs on ejaculatory latency (14). Regarding FSFI as a primary outcome of sexual dysfunction, we did not observe any difference in basal sexual function in our patients (*p* = 0.89). Although a reduction in FSFI was seen in both groups after 1 month, no significant difference was detected between the two groups. A limitation in the number of evaluated patients may have prevented us from detecting a difference between the two groups, because there are some reports regarding the variability of sexual dysfunction associated with different SSRIs. For instance, a recent cross-sectional study among 100 patients attending a university or private psychiatric clinic reported sexual dysfunction in 100% of patients who received fluoxetine and 71.4% who took citalopram (23).

We tried to exclude all confounding factors, such as lactation and pregnancy, which may have altered oxytocin levels during the study. The pattern of oxytocin levels in the circulation was the same during both stages of the menstrual cycle. Also, there was no pulsatile pattern of oxytocin level in the blood of women in the basal state (24). In this study, oxytocin level did not differ between the two groups (*p* = 0.71) at baseline, but a meaningful decrease in oxytocin level and an increase in oxytocin level was detected in our patients in the fluoxetine and citalopram groups, respectively, after 1 month (*p* = 0.05). In agreement with our study, a study by Cantor *et al*. showed that administration of oxytocin may help to alleviate the sexual side effects of fluoxetine in rats (25). We also observed a positive relationship between FSFI score and oxytocin level 1 month after starting fluoxetine or citalopram (r = 0.43, *p* = 0.04). This means that sexual functioning in the study subjects may have been improved by increasing their oxytocin levels. Not only have sexual side effects of SSRIs been attributed to oxytocin but there is also some evidence regarding the role of oxytocin in the SSRI effect. Emilliano *et al*. reported that the therapeutic effects SSRIs on social affiliation and anxiety may be mediated in part by components of oxytocin (26). 

This is the first pilot clinical trial to study the effect of two different SSRIs (fluoxetine and citalopram) on oxytocin levels in women. We also noticed, for the first time, a correlation between FSFI (as an indicator of sexual dysfunction) and oxytocin. We were limited in both the sample size and duration of the study. The patient follow up was of limited duration; thus, the comparative effect of fluoxetine and citalopram on oxytocin over the long term is unknown. 

## Conclusion

A positive relationship between the oxytocin level and FSFI score supports the hypothesis that the oxytocin level plays a role in sexual dysfunction induced by SSRIs.
